# Cross sectional study of childhood obesity and prevalence of risk factors for cardiovascular disease and diabetes in children aged 11–13

**DOI:** 10.1186/1471-2458-9-86

**Published:** 2009-03-24

**Authors:** Anwen Rees, Non Thomas, Sinead Brophy, Gareth Knox, Rhys Williams

**Affiliations:** 1 Cardiff School of Sport, University of Wales Institute Cardiff, Wales, UK; 2 School of Medicine, Swansea University, Wales, UK

## Abstract

**Background:**

Childhood obesity levels are rising with estimates suggesting that around one in three children in Western countries are overweight. People from lower socioeconomic status and ethnic minority backgrounds are at higher risk of obesity and subsequent CVD and diabetes.

Within this study we examine the prevalence of risk factors for CVD and diabetes (obesity, hypercholesterolemia, hypertension) and examine factors associated with the presence of these risk factors in school children aged 11–13.

**Methods and design:**

Participants will be recruited from schools across South Wales. Schools will be selected based on catchment area, recruiting those with high ethnic minority or deprived catchment areas. Data collection will take place during the PE lessons and on school premises. Data will include: anthropometrical variables (height, weight, waist, hip and neck circumferences, skinfold thickness at 4 sites), physiological variables (blood pressure and aerobic fitness (20 metre multi stage fitness test (20 MSFT)), diet (self-reported seven-day food diary), physical activity (Physical Activity Questionnire for Adolescents (PAQ-A), accelerometery) and blood tests (fasting glucose, insulin, lipids, fibrinogen (Fg), adiponectin (high molecular weight), C-reactive protein (CRP) and interleukin-6 (IL-6)). Deprivation at the school level will be measured via information on the number of children receiving free school meals. Townsend deprivation scores will be calculated based on the individual childs postcode and self assigned ethnicity for each participating child will be collected. It is anticipated 800 children will be recruited. Multilevel modeling will be used to examine shared and individual factors associated with obesity, stratified by ethnic background, deprivation level and school.

**Discussion:**

This study is part of a larger project which includes interviews with older children regarding health behaviours and analysis of existing cohort studies (Millennium cohort study) for factors associated with childhood obesity.

The project will contribute to the evidence base needed to develop multi-dimensional interventions for addressing childhood obesity.

## Background

The World Health organisation (WHO) predicts that by 2015 approximately 2.3 billion adults will be overweight and more than 700 million will be obese. In 2005, at least 20 million children under the age of 5 were overweight [1]. The UK, like many other countries is experiencing a massive growth in the collective weight gain of its population. Obesity is a global problem that is rising at an uncontrollable rate. Obesity can be caused by many factors, including genetics. However, the underlying cause has been attributed to two modifiable behavioural factors: food consumption and physical activity [2].

Obesity can lead to many other health complications, including hypercholesterolemia and hypertension, and this can lead to serious health consequences. CVD and diabetes are two chronic diseases which are rapidly increasing globally.

Even though the health consequences of obesity are most commonly seen during adulthood, the underlying factors of these diseases could originate during childhood. Evidence is now emerging that obesity-driven type 2 diabetes might become the most common form of diabetes in adolescents within the next ten years [3]. It is therefore vital to know exactly how early the health consequences and risk factors for these serious diseases occur, and how early the can be detected if they are to be addressed successfully.

In recent years, childhood obesity has become a world wide issue with increasing poor dietary habits together with inactivity being associated with rising levels of obesity in children. Many studies including the Muscatine Study [4,5] and the Bogalusa Heart Study [6] have convincingly shown that overweight and obesity during adolescence is a determinant of a number of CVD risk factors in adulthood. Results from longitudinal studies such as the Bogalusa Heart Study have shown that adolescent adiposity tracks moderately into adulthood, implying that preventing obesity during childhood may be advantageous to health in later life [7]. Patterns of fat distribution have also been shown to influence CVD risk. It has been discovered by many studies that abdominal obesity better predicts CVD risk compared to overall obesity [8,9]. It seems that the elevated risk of abdominal obesity is related to the visceral fat that is stored around the internal organs [10].

### Identifiable Risk Factors

Many risk factors have been recognised as contributors towards the development of CVD. These include unhealthy diets, physical inactivity, obesity, hypertension and hypercholesterolemia [11-13]. More recently other risk factors have been shown to contribute towards the development of CVD, notably, elevated concentrations of fibrinogen (Fg), C-reactive protein (CRP), interleukin-6 (IL-6); and reduced levels of adiponectin (high molecular weight). Fibrinogen is the main coagulation protein in plasma and thus promotes activities such as platelet aggregation and increased blood viscosity. Elevated fibrinogen levels have been found in obese individuals [14] and thus may have an indirect effect on CVD through levels of adiposity. Fg is also thought to be responsible for changes in other acute phase proteins, notably CRP. These are released as a result of active inflammation (an underlying cause of CVD), moreover, it appears that the inflammatory cytokine IL-6 is the underlying stimulator of these acute phase proteins. Adiponectin is a protein hormone that regulates the metabolism of lipids. It is the most abundant hormone released from fat cells and has been suggested to be anti-inflammatory [15]. Concentrations of this protein have been found to be low in obese individuals, thus contributing to the pathogenesis of CVD and increased inflammation [16]. It can therefore be seen that CVD risk may not solely be due to one factor but a combination of many which may originate through behavioural and lifestyle factors.

### Socioeconomic status, ethnicity and other factors

It has also emerged that CVD risk may differ between people of differing socioeconomic status (SES), this is true for both developed, and developing countries. In developed countries, epidemiological evidence illustrates that SES is inversely linked with CVD morbidity and mortality [17]; however, evidence of this relationship in developing countries, is sparse. This may suggest that behavioural factors have a higher influence on disease risk factor profiles that genetic factors [18-20].

As previously highlighted, there is growing evidence that the risk for CVD originates during childhood, indeed, research has found that a very low or very high birth weight is associated with increased risk of CVD [21]. Furthermore, there is evidence that birth weight varies according to ethnic background and thus individuals with Asian or African origins could be at a higher genetic risk of developing obesity and CVD [22-24].

This protocol outlines the methods to examine lifestyle and behavioural factors associated with childhood obesity, diabetes and CVD risk in school children aged 11–13 years.

## Methods and design

### Aims and objectives

The aim of the proposed project is to examine the prevalence of risk factors for CVD and diabetes, and environmental determinants associated with these risk factors among children aged 11–13.

### Primary objective

To estimate the prevalence of obesity, hypercholesterolaemia and hypertension stratified by, ethnic minority group, socioeconomic status, and school.

### Secondary objective

To examine determinants associated with increased risk factors for CVD and diabetes such as low fitness levels, high fat diet, low physical activity levels, birth weight, and family history of CVD and diabetes.

### Design

#### Recruitment

The study population will be recruited from schools in both the Swansea and Cardiff areas of South Wales. Recruitment of the schools will be based upon high ethnic minority catchment area. Subsequently, a member of the team will attend year 7 (aged 11–12) and year 8 (aged 12–13) school assemblies to give a short presentation explaining the basics of the study. An explanation of the protocol will be provided, along with the advantages of participating and the feedback that will be provided following the completion of the testing. Each child will be provided with an envelope containing an information letter, consent and assent forms, and a short questionnaire. They will be asked to read through these with their parents and if they wish to take part in the study, they will be asked to provide both their assent and their parents/guardians consent. This questionnaire includes questions about birth weight and family history of diseases such as diabetes and CVD. Parents are asked to help their child complete the questionnaire. Once these have been completed testing will begin, however, every child will be asked to verbally confirm his or her consent prior to each session, and reminded of their right to withdraw from the study at any stage. Ethics approval for this study was granted by the Local NHS Research Ethics Committee.

#### Testing procedures

Following the collection of these completed documents, all information will be inputted onto an excel spreadsheet. To help recruit further participants, leaflets will be distributed to parents/guardians via children to inform them of the importance of an active and healthy lifestyle, and these will continued to be distributed throughout the first week of testing. All testing procedures will take place during the Physical Education (PE) lessons and on school premises.

#### Anthropometrical data

Data collected are outlined in Table 1. For all anthropometrical variables (height, weight, skinfold, neck, waist and hip circumference), participants will wear PE clothing (shorts and t-shirt) with no footwear. Body weight will be measured to the nearest 0.1 kg using calibrated electronic weighing scales (Seca 770, Digital Scales, Seca Ltd, Birmingham, UK). Participants will be asked to step onto the scales and wait for 3 seconds whilst the scales determine the correct weight. Stature will be measured using a portable stadiometer (Seca Stadiometer, Seca Ltd, Birmingham, UK). Participants will be asked to stand with their backs straight, feet flat and arms hanging loosely by their sides. Stature will be measured as the maximum distance from the floor to the vertex of the head and recorded to the nearest millimetre. Body mass index (BMI) will then be calculated using the BMI formula of dividing the participant's weight by their height squared [25]. This will be recorded and compared to national guidelines [26] to establish whether a participant falls into a healthy weight, underweight, overweight or obese category. Biceps, triceps, subscapular and suprailiac skinfold measurements will be taken on the right side of the body using Harpenden skinfold callipers (John Bull, British Indicators Ltd, West Sussex, UK). Participants will be asked to stand with a relaxed arm for both the triceps and subscapular measurement, a relaxed arm with palm facing forward for the bicep measurement and with their arm crossed horizontally across their chest for the suprailiac measurement. In order to take the skinfold measurements, the researcher will grasp the skin at the necessary site and place the callipers around the skin. The researcher will remain to hold the skin whilst the callipers are held for 3 seconds and the reading determined. Duplicate measurements will be taken at each site and the average recorded. The sum of these four skinfold measurements will then be calculated and recorded. Neck, waist and hip circumference will be measured on each participant and recorded to the nearest millimetre using a standard flexible measuring tape (Rosscraft, UK). These will provide an index of fat distribution [27]. Waist circumference will be measured at the narrowest part of the trunk whilst the hip circumference will be measured around the maximum protrusion of the buttocks [28].

**Table 1:** Data collected

**Individual Level Data**	Data
Questionnaire	Name, date of birth, birth weight, school, medical history of child, GP; family history of obesity – related conditions; parental height, weight, waist circumference, postcode and DOB.
Anthropometrical data	Height, weight (BMI), Waist, hip and neck circumference, percentage body fat as assessed by skinfold thickness
Physiological measurements	Blood pressure, 20 metre shuttle run
Blood tests	Cholesterol, fasting triglyceride, insulin, glucose, fibrinogen, leptin, adiponectin
Dietary questionnaire	Seven day food diary
Physical activity	Self reported activity levels on Physical Activity Questionnaire, Accelerometer.
Ethic group	Caucasian, South Asian, African or other.
	
**School Level Data**	
Deprivation	Proportion registered for free school dinners.

#### Physiological measurements

Systolic and diastolic blood pressure (BP) will be measured using an Omron M6 automatic BP monitor (Omron Healthcare UK Ltd, Milton Keynes, UK). Blood pressure (BP) will be taken after each participant has sat quietly for 10 minutes. All BP measurements will be taken by the PR to ensure consistency. The cuff will be positioned tightly on the upper left arm and the machine started. BP will be taken three times and the average of the second and third readings will be recorded for analysis [29].

Aerobic fitness will be measured using the 20 metre multi stage fitness test (20 MSFT). This field test has been validated as a predictor of maximal aerobic power in young people [30] and has been found to be a consistent and reliable field test of aerobic fitness [31]. The test requires the participants to run between two cones that have been set 20 m apart while keeping a pace with a pre-recorded auditory signal. The initial running pace is set at 8.5 km/hr and increases by 0.5 km/hr with each subsequent level. Each level is announced by the tape which is produced by the National Coaching Foundation. Participants will receive verbal encouragement from the researchers throughout the test. Once participants have reached their maximal effort and withdrawn from the test, the number of shuttles will be recorded.

#### Measures of deprivation and ethnicity

The school will be asked to complete a form providing information on the number of children receiving free school meals. This will be used as a measure of deprivation at the school. Each child will also be asked to report their postcode. Subsequently, a Townsend score of fifths of deprivation will be assigned to each individual child based on the postcode of their home address. Each child will be requested to self assign their ethnicity and this will be recorded during the anthropometrical data collection sessions.

#### Physical activity

Each participant will be asked to complete the physical activity questionnaire for adolescents (PAQ-A). The questionnaire is a seven day recall on physical activity; a validated questionnaire that has been used widely in research [32,33]. The questionnaire will be administered and completed during the same session as the BP. Instructions will be explained to participants prior to starting the test and they will be encouraged to complete the questionnaire alone. It will take approximately 20 minutes to complete the questionnaire.

#### Dietary intake

Daily food intake will be assessed using a validated, self reported seven day food diary [34]. Participants will be asked to complete this diary at home and record what was consumed for breakfast, lunch and dinner and snacks. It will take approximately 5 minutes to complete per day. A short dietary questionnaire will also be included, and this will complement the food diary. The food diary will be analysed by Health Options Ltd (Health options Ltd, Cirencester, Gloucester, UK). Average daily kilojoules, percentage of total fat, saturated fat, carbohydrate, protein and fibre will be calculated.

#### Lipids and lipoproteins

Venous blood samples will take place during the morning between 9 am and 10.30 am, following an overnight fast and after the participants have sat quietly for 30 minutes. Blood samples will be taken by qualified phlebotomists and a health professional (nurse or doctor) will be present at all times. The sampling will take place in the school gymnasium or a suitable area that will be divided into cubicles as necessary by screens. Participants will be called in alphabetical order and accompanied by one of the researchers. Whilst participants are waiting their turn, a suitable DVD will be shown. Immediately following sampling, the participants will be provided with breakfast in the school canteen.

Blood samples will be drawn to measure levels of glucose, insulin, lipids, fibrinogen (Fg), adiponectin (high molecular weight), C-reactive protein (CRP) and interleukin-6 (IL-6).

#### Feedback

On completion of all data collection, feedback will be provided to the school and participants on both an individual and group basis. Each participant will receive a full breakdown of their results with suitable advice to help modify their lifestyle if needed. Both parents and family doctor are informed of any abnormal findings. Group feedback will be presented to the headmaster and PE staff.

#### Analysis Plan

A multi-level analytical approach will be used to examine the relationship between outcomes (obesity, hypertension, hypercholesterolemia), and individual and group level determinant variables. This approach overcomes common methodological barriers associated with conventional regression analysis in epidemiology, where correlation among individuals sharing the same local environment is not accounted for. Multilevel modelling allows for the examination of variability in outcomes between individuals as well as between higher level units.

The overall levels of obesity, hypertension and hypercholesterolemia will be examined both on the crude level, and stratified by ethnic background and deprivation. Factors associated with obesity, and separately with hypertension and hypercholesterolemia will be assessed using multilevel modelling accounting for shared environment at the school level and neighbourhood level. Factors examined will include; birth weight, family history of obesity -related conditions, fitness tests, risk factors in the blood (insulin, glucose, fibrinogen, leptin, adipondectin), dietary analysis, deprivation, school, physical activity levels, ethnic group, fitness tests, percentage body fat, waist/hip ratio.

#### Sample size

This study aims to recruit five schools with participation approximating 150–200 children in each school (n = 800). A total sample size of 800+ will give prevalence estimates of obesity within 3% accuracy. Data collected in this study will be combined and compared with the 400 children collected using the same methods in Carmarthenshire in 2000 and 2006–2007. This will provide a total sample size of 1200 children.

#### Handling of missing and incomplete data

The fitness levels and sex of the participants in each school will be compared with the class averages. This will provide an estimate of the generalisability of the findings in each school according to the class they represent. Where participants are found to differ from the class average, weighted scores will be presented along with crude prevalence figures.

Missing data for individuals will be imputed using measures on individuals with comparable scores in other known values. For example, waist measurement may be imputed from a different participant with the same BMI and height. Results based on data without and with imputed fields will be presented.

## Discussion

This study will provide estimates of obesity levels by ethnic group and socio-economic status for children aged 11–13 years. It will offer an evidence base to identify those children at high risk of obesity and future health problems in order to inform and target interventions to address childhood obesity.

## Abbreviations

BMI: Body Mass Index; CVD: Cardiovascular Disease; PAQ: Physical Activity Questionnaire; CRP: C-reactive protein; IL-6: Interlerleukin-6; MSFT: Multistage fitness test; Fg: fibrinogen; SES: socioeconomic status; BP: Blood Pressure.

## Competing interests

The authors declare that they have no competing interests.

## Authors' contributions

NT and AR designed and wrote the original proposal. This as been modified and adapted by SB, GK and RW.

## Pre-publication history

The pre-publication history for this paper can be accessed here:


